# Transitioning from Clinical Trial to Clinical Practice for Long‐Term Lecanemab Treatment in Early Alzheimer’s Disease: Perspectives from an Alzheimer’s Disease Treatment Center

**DOI:** 10.1002/alz70859_101400

**Published:** 2025-12-25

**Authors:** David Watson, Michael G Neam, Mark Stafford, Cynthia Bouchard, Venessa Ranney, Sydney Werner

**Affiliations:** ^1^ Alzheimer's Research and Treatment Center, Wellington, FL USA; ^2^ Alzheimer’s Research and Treatment Center, Wellington, FL USA

## Abstract

**Background:**

Lecanemab, an anti‐amyloid monoclonal antibody, reduced amyloid markers in early Alzheimer’s disease (AD) and slowed cognition and function decline in the phase 2 Study 201 and phase 3 Clarity AD trials at 18 months, while maintaining those benefits longer‐term in the ongoing open‐label extension (OLE) study. Herein, we provide our experience with 136 individuals with early AD treated at our center as part of lecanemab clinical trials.

**Methods:**

We conducted a single‐center, retrospective case series investigation in people with confirmed early AD treated with lecanemab at the Alzheimer’s Research and Treatment Center (Wellington, Florida). Patients had been initiated on lecanemab therapy as part of Study 201 or Clarity AD trials, and then continued long‐term lecanemab in our center. Data collection included baseline characteristics, treatment exposure, efficacy and safety. Patients and care partner perspectives have been evaluated via a patient/caregiver experience telephone survey based on What Matters Most concepts.

**Results:**

136 people were randomized to either lecanemab or placebo in either Study 201 (lecanemab:53; placebo:16) or Clarity AD (lecanemab:37; placebo:30). Overall, 42 patients with ≥3 years on lecanemab, including 30 with ≥4 years and 14 with ≥5 years exposure. Baseline characteristics were similar at our center and in the clinical trials. In Study 201 patients, there was continuous amyloid reduction over 4 years and meaningful delays in time‐to‐progression to the next stage of AD. These results are consistent with Clarity AD OLE 36 months data. No new safety signals were observed for individuals at our center, with no treatment discontinuations due to adverse events. Adverse events, including ARIA, were consistent with the published lecanemab safety profile. Responses from patients and care partners with >5 years treatment included stabilized disease, slowing of disease progression, and quality‐of‐life improvement.

**Conclusion:**

Long‐term lecanemab safety/efficacy profile with our real‐world experience was similar to that observed in clinical trials. Continued lecanemab treatment beyond the double‐blind trials supports meaningful delay in disease progression. Most patients in the clinical trials elected to stay on long‐term lecanemab therapy and believe the therapy is extending/improving their lives.

